# Analysis of HMGCS2 Expression and TG Lipidomics in the Perirenal Adipose Tissue of Obese Diabetic Nephropathy Mice

**DOI:** 10.1111/1753-0407.70125

**Published:** 2025-07-25

**Authors:** Yuhong Huang, Miao Zeng, Mengxue Yang, Xiaodi Zheng, Lulu Jin, Rui Zhang, Yueyue Wu, Fei Li, Bo Yang, Jun Liu

**Affiliations:** ^1^ Department of Endocrinology Shanghai Fifth People's Hospital Affiliated to Fudan University Shanghai China; ^2^ Fudan University Community Health Research Center (To Be Established) Shanghai China; ^3^ Department of Infectious Diseases Shanghai Fifth People's Hospital Affiliated to Fudan University Shanghai China; ^4^ Department of Endocrinology Shanghai Pudong New Area People's Hospital Shanghai China; ^5^ Department of Endocrinology Affiliated Hospital of Zunyi Medical University Zunyi China

**Keywords:** diabetic kidney disease, HMGCS2, lipidomics, perirenal adipose tissue (PRAT), TG molecules

## Abstract

**Background and Aims:**

Renal HMGCS2 upregulation is associated with lipid deposition. However, the expression pattern and role of Hmgcs2 in the perirenal adipose tissue (PRAT) is not clear. This study was designed to elucidate the contribution of Hmgcs2 in the pathogenesis of obese diabetic kidney disease mice.

**Methods:**

12‐week‐old db/db (diabetic) and db/m (control) mice were fed high‐fat or normal diets, respectively. At 12, 16, and 20 weeks, mice (*n* = 4/group/timepoint) were euthanized for metabolic profiling (body weight, blood glucose, urinary ACR) and tissue collection (kidney, PRAT). Tissues were analyzed for TNF‐α mRNA (qPCR), HMGCS2 expression (IHC/WB/IF), lipid deposition (Oil Red O), and histopathology (HE staining). PRAT triglycerides (colorimetric assay) and lipidomics (UPLC–MS/MS) were assessed. HMGCS2‐knockout mice (CRISPR‐generated) underwent metabolic tests (OGTT/ITT) before terminal tissue analysis.

**Results:**

(1) Compared with db/mPRAT, db/db PRAT had significantly enlarged adipocytes and increased TG content. The expression of HMGCS2 in renal and PRAT was significantly greater in db/db mice. (2) Hmgcs2 was equally expressed in db/db renal and PRAT. PRAT expansion increases the inflammatory factor TNF‐α, which occurs earlier in PRAT than in renal tissue.(3) Genetic ablation of HMGCS2 in mice significantly decreased renal and PRAT TG accumulation, concomitant with attenuated inflammation. (4) LC–MS/MS analysis revealed that TGs are the main PRAT lipid component. Db/db PRAT TG content was significantly greater than that in db/m. Db/db proximal PRAT TG content is greater than that of the distal region, with seven upregulated TG lipid molecules (TG (38:3)+NH4, TG (50:5)+NH4, TG (52:12e)+Na, TG (56:9e)+H, TG (57:6e)+H, FA (18:1)+H, and ST (m45:3)+NH4), among which TG (38:3) has the highest expression.

**Conclusion:**

Our study strongly suggests that lipids, especially TGs, are deposited in the kidneys and PRAT of DKD mice, with proximal–distal PRAT differences. HMGCS2 may be involved in kidneys and PRAT TG deposition. PRAT‐lipid‐metabolism‐induced inflammation may occur before blood‐glucose‐related kidney damage.

## Graphical abstract

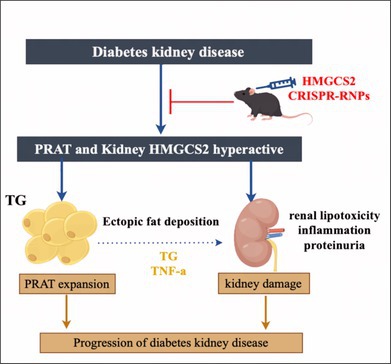

**Summary**

- What is already known about this subject
  ○ With DKD, lipid deposition increases in perirenal tissue.
  ○ HMGCS2 expression increases in DKD renal tissue owing to triglyceride deposition. However, research on HMGCS2 in PRAT‐DKD has not been reported.
- What is the key question?
  ○ What role does HMGCS2 play in PRAT lipid deposition in DKD?
- What are the new findings
  ○ The expression of HMGCS2 is upregulated in the kidneys and PRAT of DKD mice.
  ○ Inflammation caused by lipid metabolism in PRAT may occur before the damage to the kidneys caused by blood glucose, and there are differences in lipid metabolism between proximal and distal PRAT.
- How might this impact clinical practice in the foreseeable future?
  ○ PRAT and HMGCS2 may be potential therapeutic targets for early intervention of diabetic nephropathy.

**Abbreviations**
AcAc‐CoA: acetoacetyl‐CoA
CPROBE: clinical phenotyping and resource biobank core
DKD: diabetic kidney disease
ESRD: end‐stage renal disease
FAO: fatty acid oxidation
FFA: free fatty acids
HDAC: histone deacetylase
HIF‐α: inducible factor‐α
HMGCL: hydroxy‐methyl‐glutaryl‐CoA lyase
HMG‐CoA: 3‐hydroxy‐3‐methylglutaryl‐CoA
HMGCS2: 3‐hydroxy‐3‐methylglutaryl‐CoA synthase 2
IHC: immunohistochemical
MS: mass spectrometry
PRAT: perirenal adipose tissue
PRFT: perirenal fat thickness
ROS: reactive oxygen species
SREBPs: sterol regulatory element‐binding proteins
T2DM: type 2 diabetes mellitus
TG: triglyceride
UPLC: ultra performance liquid chromatography

## 1 Introduction

Diabetic kidney disease (DKD), a serious complication of diabetes, is the leading cause of chronic kidney disease [1]. Currently, various treatment strategies, including angiotensin‐converting enzyme inhibitors (ACEIs), angiotensin II receptor blockers (ARBs), and sodium‐dependent glucose transporter 2 (SGLT‐2) inhibitors, are available. However, DKD still inevitably progresses to end‐stage renal disease (ESRD) even after treatment. Among ESRD patients who start dialysis, the five‐year survival rate does not exceed 60% [2]. Therefore, new kidney protection strategies are urgently needed.

Perirenal adipose tissue (PRAT) is a fat pad that surrounds the kidneys and has a complete blood supply and innervates nerves and lymphatic vessels. A previous study suggested that PRAT is involved in chronic kidney injury and is an independent risk factor for DKD in T2DM patients [3]. Multiple studies have shown that the PRAT thickness (PRFT) of patients with kidney injury is significantly greater than that of individuals with normal kidney function. Moreover, PRAT is negatively correlated with the eGFR. With the deposition of PRAT, the risk of proteinuria in T2DM patients increases 19.3‐fold, and the severity of DKD increases accordingly [4, 5, 6]. In addition to its mechanical effects, insulin resistance, and overactivation of the RAAS, the possible mechanism by which PRAT induces DKD involves free fatty acid accumulation, adipokine secretion, and oxidative stress.

Dysregulation of lipid metabolism is associated with kidney damage, and obesity is an independent risk factor for chronic kidney disease. Obesity‐induced lipid metabolism disorders, especially FFA (free fatty acids) entering the kidneys, cause fat accumulation, lipid toxicity, and inflammatory reactions. The specific mechanism is that the upregulation of SREBP‐1 promotes the synthesis of fatty acids and triglycerides (TG) in the kidneys [7]. However the precise mechanistic links between perirenal adipose tissue (PRAT) lipid dysregulation and DKD progression remain incompletely understood.

Fatty acid oxidation is a primary energy source for the kidneys. 3‐Hydroxy‐3‐methylglutaryl‐CoA synthase 2 (HMGCS2) is the first rate‐limiting enzyme for the production of ketone bodies during fatty acid beta oxidation in mitochondria [8]. Two acetyl‐CoA molecules are catalyzed by acetyl‐CoA thiolase to form acetoacetyl‐CoA (AcAc‐CoA), which is then catalyzed by HMGCS2 to form 3‐hydroxy‐3‐methylglutaryl‐CoA (HMG CoA). HMG‐CoA is cleaved by HMG‐CoA lyase (HMGCL) to participate in ketogenesis, while also serving as an intermediate in cholesterol biosynthesis. Elevated HMGCS2 expression indicates enhanced fatty acid degradation and ketone body production, while potentially increasing lipogenesis. Ketones can increase cellular oxidative stress, increase reactive oxygen species (ROS), cause lipid peroxidation, epithelial cell mesenchymal differentiation, chronic inflammation and fibrosis, and accelerate the progression of DKD [9]. Bai et al. [10] upregulated the expression of renal HMGCS2 leading to the progression of DKD. Afterward, HMGCS2 expression decreased, and renal fibrosis was alleviated. These findings suggest that HMGCS2 can serve as a new potential therapeutic target for DKD. Currently, research on HMGCS2 in CKD has focused mainly on its expression in renal tissue, but there are no reports on the expression of HMGCS2 in PRAT.

As lipids are deposited, PRAT expands and causes circumscribed tissue hypoxia. The hypoxic environment triggers the release of hypoxia inducible factor‐α (HIF‐α), which can stimulate macrophages to secrete proinflammatory factors, such as TNF‐α and IL‐6, triggering inflammatory reactions within PRAT [11, 12]. In our pre‐experiment, we found that HMGCS2 expression increases in the PRAT of DKD mice while lipid deposition increases. Therefore, we speculate that HMGCS2 may be involved in the deposition of lipids in the kidneys and PRAT of DKD mice and exacerbate damage to the kidneys.

In this study, we focused on the role of HMGCS2 in the kidney and PRAT lipid deposition process in DKD mice. We found that the level of HMGCS2 was increased in renal and PRAT with DKD mice, especially in PRAT. The upregulation of HMGCS2 in PRAT was associated with lipid accumulation. HMGCS2 knockdown in mice reduced TG content in PRAT, attenuated renal inflammation, and decreased urinary protein levels. Our findings indicate that HMGCS2 may represent an attractive biomarker as well as a novel therapeutic target for DKD.

## 2 Methods

### 2.1 Animal Model and Experimental Groups

All operations were carried out in accordance with the National Institute of Health (NIH) guidelines for the Care and Use of Laboratory Animals [13] and under the approval offered by the Ethics Committee of the Veterinary Drug Evaluation Center of the Shanghai Veterinary Research Institute, Chinese Academy of Agricultural Sciences (Ethics Approval SV‐20230908‐07).

#### 2.1.1 Experimental Groups

DKD group (db/db mice) and CON group (db/m mice).

#### 2.1.2 Experimental Animals

Twelve week old male db/db mice (*n* = 16) and db/m mice (*n* = 16) (purchased from Beijing Huafukang Biotechnology Co. Ltd., license number: SCXK [Beijing] 2020‐0004) were selected.

### 2.2 Dietary Interventions

Db/db mice (weight 32–36 g) were fed a high‐fat diet (HFD; 60% kcal fat, Jiangsu Medison Biopharmaceutical Co. Ltd.), while db/m mice (20–27 g) received a normal diet (ND). All mice were maintained in specific pathogen‐free (SPF) facilities with controlled environmental parameters (temperature: 22°C ± 1°C, humidity: 55% ± 5%, 12‐h light/dark cycle).

### 2.3 Experimental Design

Body weight, fasting blood glucose, and urinary albumin‐to‐creatinine ratio (ACR) were assessed at 12, 16, and 20 weeks of age. At each time point (*n* = 4 mice/group/time point; total *n* = 12/group), animals were humanely euthanized for tissue collection (serum, kidneys, and PRAT). The remaining mice were maintained until terminal analysis at 20 weeks, comprising: DKD model: db/db mice (*n* = 4); control: db/m mice (*n* = 4); lipidomics cohort: PRAT samples analyzed by LC–MS/MS (*n* = 4/group).

### 2.4 Generation of HMGCS2‐ko Mouse Model for DKD

Male and female HMGCS2+/− mice (C57BL/6J background; Saiye Biotechnology) were used to establish the knockout model. CRISPR/Cas9‐mediated gene editing was performed using two target‐specific sgRNAs: sgRNA‐A1 (reverse strand): 5′‐TATGGGCTTCTGATCCAGG‐3′ and sgRNA‐B1 (forward strand): 5′‐CAACTCCCTGCCTGGACAGTGG‐3′. These guides targeted exon 2 of HMGCS2, inducing a frame shift mutation that disrupted protein function. Ribonucleoprotein complexes (RNPs) containing Cas9 and sgRNAs were microinjected into fertilized zygotes. Male HMGCS2−/− homozygous mice and wild‐type (WT) littermates were selected for subsequent metabolic studies after confirmation of complete gene ablation.

HMGCS2‐ko mice (12 W, *n* = 4) received HFD for 8 weeks. Terminal collection of serum, kidneys, and PRAT was performed at 20 weeks under anesthesia.

### 2.5 Metabolic and Tissue Parameter Measurements

Following abdominal cavity exposure, perirenal adipose tissue (PRAT) was excised and weighed. PRAT mass and body weight were recorded. Tail blood glucose was measured (Sanuo Biosensing). 24‐h urine samples were analyzed for mAlb/Cr ratio (Beckman AU5821).

### 2.6 Determination of PRAT TG Content

Part of the PRAT was sent for lipid proteomic analysis, and the other part was fixed with paraformaldehyde (PFA) for later experiments. PRAT TG content was estimated via colorimetry using a commercial kit (Solarbio). At the end of the experiment, the PRAT homogenate was collected and cracked in ice‐cold n‐heptane/isopropanol (1:1) solution. Lipids were extracted by centrifugation at 10000 × *g* for 10 min at 4°C to obtain the total lipid content, and the TG content of PRAT (g/mg tissue) was measured according to the instructions of the kit.

### 2.7 TNF‐α Quantification by ELISA

Renal and PRAT samples from 20‐week‐old mice were homogenized in cold PBS (1:10 w/v) containing protease inhibitors. TNF‐α protein levels were quantified using the Mouse TNF‐α Quantikine ELISA Kit (R&D Systems, Cat# MTA00B) according to the manufacturer's protocol. Absorbance was measured at 450 nm with wavelength correction at 540 nm. TNF‐α concentrations were calculated from the standard curve and expressed as pg/mg of total tissue protein.

### 2.8 Renal Histology and H&E Staining

The preserved renal tissues were fixed in 4% PFA, paraffin embedded, and cut into 5 μm‐thick slices. The samples were deparaffinized and hydrated and then further subjected to PAS via periodic acid oxidation and Schiff's reagent staining. Hematoxylin and eosin were used to stain the slices for H&E staining, which was subsequently used for pathological evaluation of the kidneys. Then, the slices were mounted with neutral gum. An optical microscope was used to obtain micrographs at a magnification of 200× (Leica, DM2500, Wetzlar, Germany).

### 2.9 Immunohistochemical (IHC) Staining

The renal tissue sections were dewaxed and dehydrated. After being washed with PBS and blocked, the slices were incubated with the primary antibody HMGCS2 (1:2000) overnight at 4°C. The samples were subsequently incubated with secondary goat anti‐mouse or goat anti‐rabbit IgG antibodies (1:250, Servicebio) for 1 h. After washing with PBS, all the sections were subjected to DAB staining and imaged via a light microscope. Nuclei stained with hematoxylin appear blue, whereas those stained brown indicate HMGCS2 positivity. Optical density analysis was carried out with the software Image‐Pro Plus v 5.1.

### 2.10 RNA Extraction and Quantitative qPCR

Total RNA was extracted via TRIzol Reagent. TRIzol reagent was used following the instructions provided by the manufacturer. After being diluted, total RNA (2 μg) was subjected to reverse transcription with Minami RNA cDNA reverse transcription kits (TaKaRa, Dalian, China). To determine the relative mRNA level, qPCR was performed using HMGCS2 Master Mix, and gene expression was normalized to that of GAPDH. The primers used were designed and synthesized by Sangan Biotech (Shanghai, China), and the sequences are listed in Table 1.

**TABLE 1:** The sequences of primers.

Gene	Forward (5′–3′)	Reverse (5′–3′)
TNF‐a	ATAGCTCCCAGAAAAGCAAGC	CACCCCGAAGTTCAGTAGACA
GAPDH	GCCTCCTGCACCACAAACT	GGACCATCCACGGTCTTCT

### 2.11 Western Blotting

Murine renal tissue was lysed with radioimmunoprecipitation assay (RIPA) lysis buffer, and total protein was extracted from the tissue supplemented with protease inhibitors and phosphatase inhibitors. The proteins were separated via SDS–PAGE and transferred to PVDF membranes. The membranes were blocked in 5% nonfat milk in TBST buffer for 2 h at room temperature, incubated with primary antibodies (HMGCS2 1:1000; GAPDH 1:5000, ß‐actin 1:5000) overnight at 4°C, and then with secondary antibodies for 1 h at room temperature, after which they were developed via an enhanced chemiluminescence (ECL) system.

### 2.12 Immunofluorescence Staining

The PRAT were perfused with PBS and fixed with 4% PFA overnight. The adipose tissue was embedded in OTC cryoculture medium and cut into 10‐μm‐thick slices. The slices were blocked with 10% bovine serum albumin (PBS) at room temperature for 1 h and incubated with the monoclonal primary antibody HMGCS2 (Code: A14244, ABclonal, China) overnight at 4°C. After washing with PBS, the slices were incubated with the secondary antibodies goat anti‐mouse IgG H&L DyLight488 (Code: 96879, Abcam) for 1 h at room temperature and counterstained with Hoechst. The images were visualized and analyzed via confocal microscopy.

### 2.13 Lipid Deposition Analysis by Oil Red O Staining

FPRAT and kidney tissues were rinsed in cold PBS, fixed in 4% PFA for 24 h at 4°C, cryo‐embedded in OCT compound, sectioned at 15 μm thickness using a cryostat, stained with 0.5% Oil Red O (in 60% isopropanol) for 15 min, counterstained with hematoxylin. Neutral lipid droplets were visualized as bright red deposits under light microscopy.

### 2.14 UPLC–MS/MS Analysis

Perirenal adipose tissue (PRAT) samples (including adjacent renal tissue) from all experimental groups were processed for comprehensive lipid analysis. Ultra Performance Liquid Chromatography (UPLC) and tandem mass spectrometry (MS/MS) analysis was performed at Paisenno Biotechnology Co. Ltd. (shanghai, China).

### 2.15 Statistical Analyses

Prism 8 software was used for analysis and mapping. SPSS 25.0 software was used for statistical analysis. The measurement data are expressed as mean (*x*) ± standard deviation (*s*). Independent‐sample *t* tests were used to compare two groups of measurement data. *p* < 0.05 was considered significant.

## 3 Results

### 3.1 Weight and TG Content Increased in PRAT of Db/Db Mice

**FIGURE 1:**
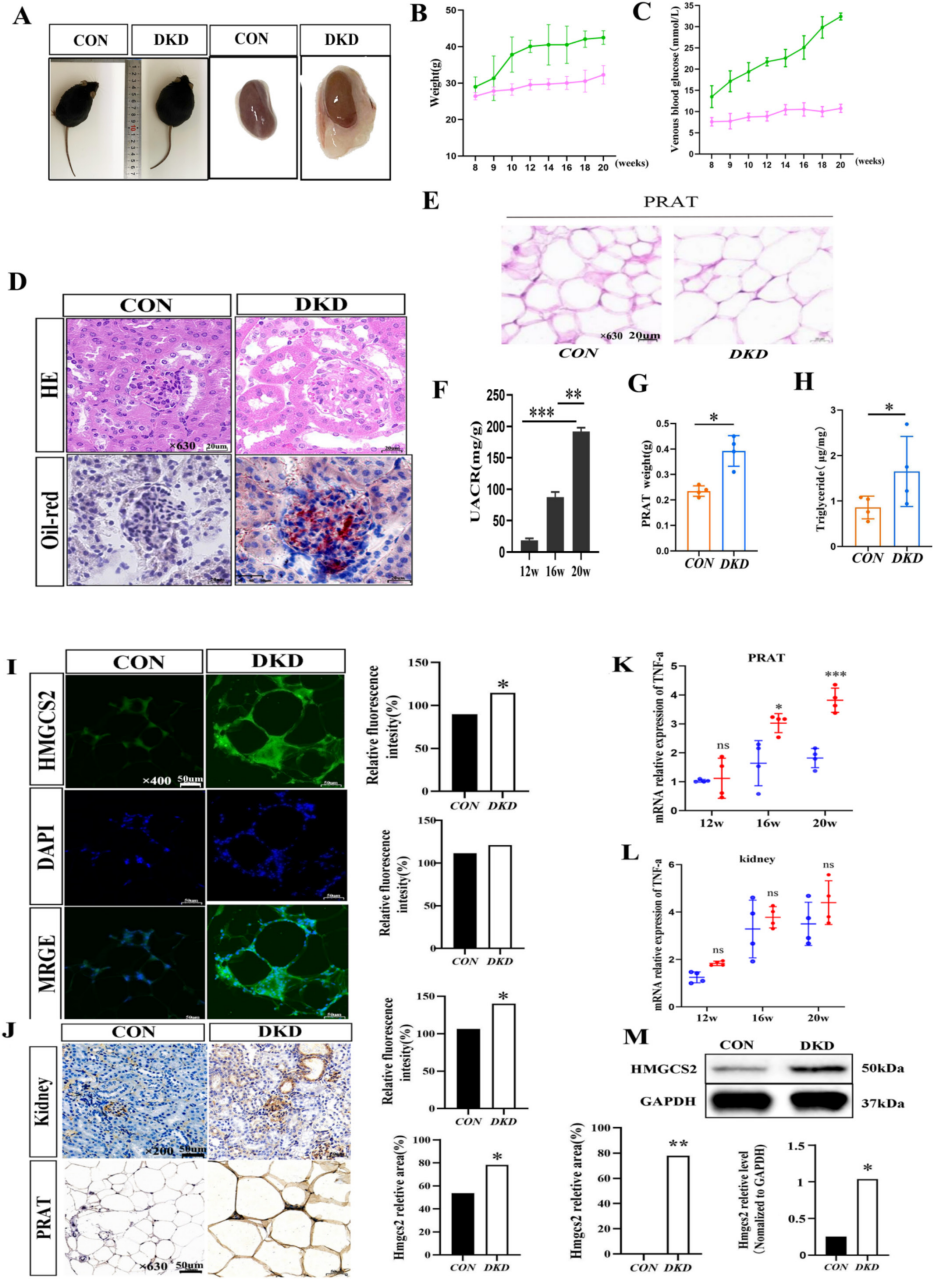
(A) The images of mice and PRAT. The body weights (B) and Fasting blood glucose (C) of two groups mice (green: *db/db*; pink: *db/m*). (D) HE staining and Oil‐red staining of murine kidney tissues from two group mice (×630), scale bar = 20 μm. (E) HE staining of PRAT in two group mice (×630), scale bar = 20 μm. (F) Mouse UACRs were tested at 12, 16 and 20 weeks. (G) weights of PRAT in two group mice. (H) TG contents of PRAT in two group mice. (I) Immunofluorescence staining and fluorescence intensity analysis of HMGCS2 in different groups of PRAT as indicated (×400), scale bar = 50 μm. (J) Immunohistochemical staining and semiquantification of HMGCS2 expression in mouse kidney and PRAT samples, scale bar = 50 μm. The relative mRNA levels of TNF‐a in different groups of PRAT (K) and kidney (L) samples were determined via qRT–PCR. Each bar represents the mean ± SD of the data derived from three independent experiments (*n* = 4). (M) Western blot analysis of HMGCS2 expression in PRAT and densitometric analysis of the blots. GAPDH served as a loading control. *p < 0.05, **p < 0.01, ***p < 0.001 versus *db/m* mice.

High‐fat diet‐fed db/db mice exhibited progressive metabolic deterioration, developing significant obesity accompanied by PRAT expansion (Figure 1A). These mice displayed hallmark diabetic features, including increased weight (Figure 1B), elevated fasting blood glucose (Figure 1C) and increased UACR (Figure 1F). Histopathological analysis revealed both glomerular hypertrophy and marked renal lipid accumulation (Figure 1D). Furthermore, DKD mice exhibited significant pathological remodeling of PRAT, characterized by adipocyte hypertrophy (Figure 1E), elevated weight (Figure 1G) and increased triglyceride accumulation (Figure 1H). These morphological and metabolic alterations collectively demonstrate substantial adipose tissue dysfunction in DKD progression.

### 3.2 HMGCS2 Was Significantly Induced in PRAT and Kidney Under Diabetic Conditions

DKD mice demonstrated significant upregulation of HMGCS2 expression in both PRAT and kidney tissues, as evidenced by immunofluorescence (Figure 1I), immunohistochemistry (Figure 1J), and Western blot analysis (Figure 1M). Notably, TNF‐α levels exhibited distinct temporal patterns, with PRAT showing early inflammatory activation at 16 weeks (Figure 1K) while kidney tissue displayed no significant difference even at 20 weeks (Figure 1L). These findings collectively suggest that HMGCS2 overexpression may contribute to DKD pathogenesis, with PRAT‐derived inflammation potentially serving as an early driver of renal injury through tissue‐specific metabolic‐inflammatory crosstalk.

### 3.3 HMGCS2 Contributed to PRAT Lipotoxicity Under Diabetic Conditions

**FIGURE 2:**
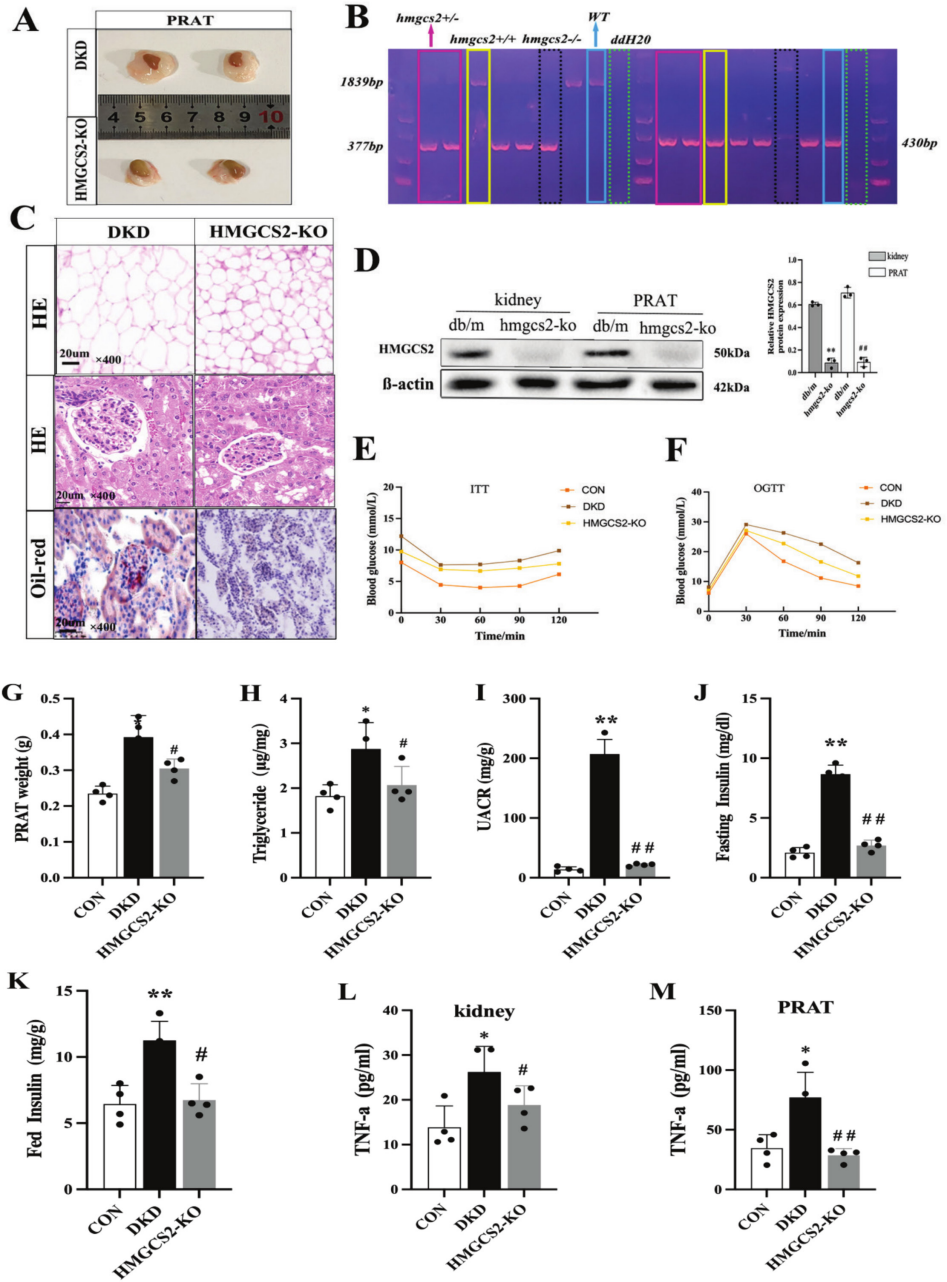
(A) The images of kidney and PRAT from two group mice. (B) Genotyping was confirmed by tail preparation and PCR at 2 weeks of age. (C) HE staining and Oil‐red staining of murine kidney and PRAT tissues from two group mice. (D) Representative Western blot and quantifications of HMGCS2 expression in kidney and PRAT from two group mice. **p < 0.01 versus *db/m*. (E) Insulin Tolerance Test. (F) Oral Glucose Tolerance Test. (G) Weights of PRAT in two group mice. (H) TG contents of PRAT in two group mice. (I) Urinary albumin creatinine ratio (UACR) in different groups of mice (*n* = 4 for each group). Fasting insulin (J) and fed insulin (K) in different groups of mice. TNF‐a protein in different groups of kidney (L) and PRAT (M) samples were determined via ELISA. *p < 0.05, **p < 0.01 versus *db/m* mice, ^#^ *p* < 0.05, ^##^ *p* < 0.01 versus *db/db* mice.

To further elucidate the role of HMGCS2 in PRAT under diabetic conditions, we generated HMGCS2 knockout mice by using CRISPR/Cas9, which were validated by tail genotyping (Figure 2B) and Western blot analysis for HMGCS2 expression in PRAT and kidney (Figure 2D). Then db/db, HMGCS2‐ko, and their controls in age‐matched groups were analyzed in this study. At 20 weeks of age, HMGCS2 deletion provides protection in DKD mice. This protection includes metabolic improvements such as reduced PRAT thickness (Figure 2A), improved glucose homeostasis (Figure 2E,F,J,K) and attenuated proteinuria (Figure 2I). Additionally, HMGCS2 knockout induces PRAT remodeling characterized by smaller adipocytes (Figure 2C), decreased PRAT weight (Figure 2G), and lower TG content (Figure 2H). Compared with db/db mice, HMGCS2‐ko mice show reduced glomerular hypertrophy by HE staining and had fewer neutral lipids deposition in glomeruli by Oil Red O staining (Figure 2C). Moreover, gene silencing of HMGCS2 reduced the levels of proinflammatory mediators (Figure 2L,M) in PRAT and kidney of HMGCS2‐ko mice. These data indicate that HMGCS2 plays a critical role in lipid deposition and inflammatory response of PRAT in DKD mice.

### 3.4 Differential Lipid Composition Analysis of PRAT in Db/Db Mice and Db/m Mice

**FIGURE 3:**
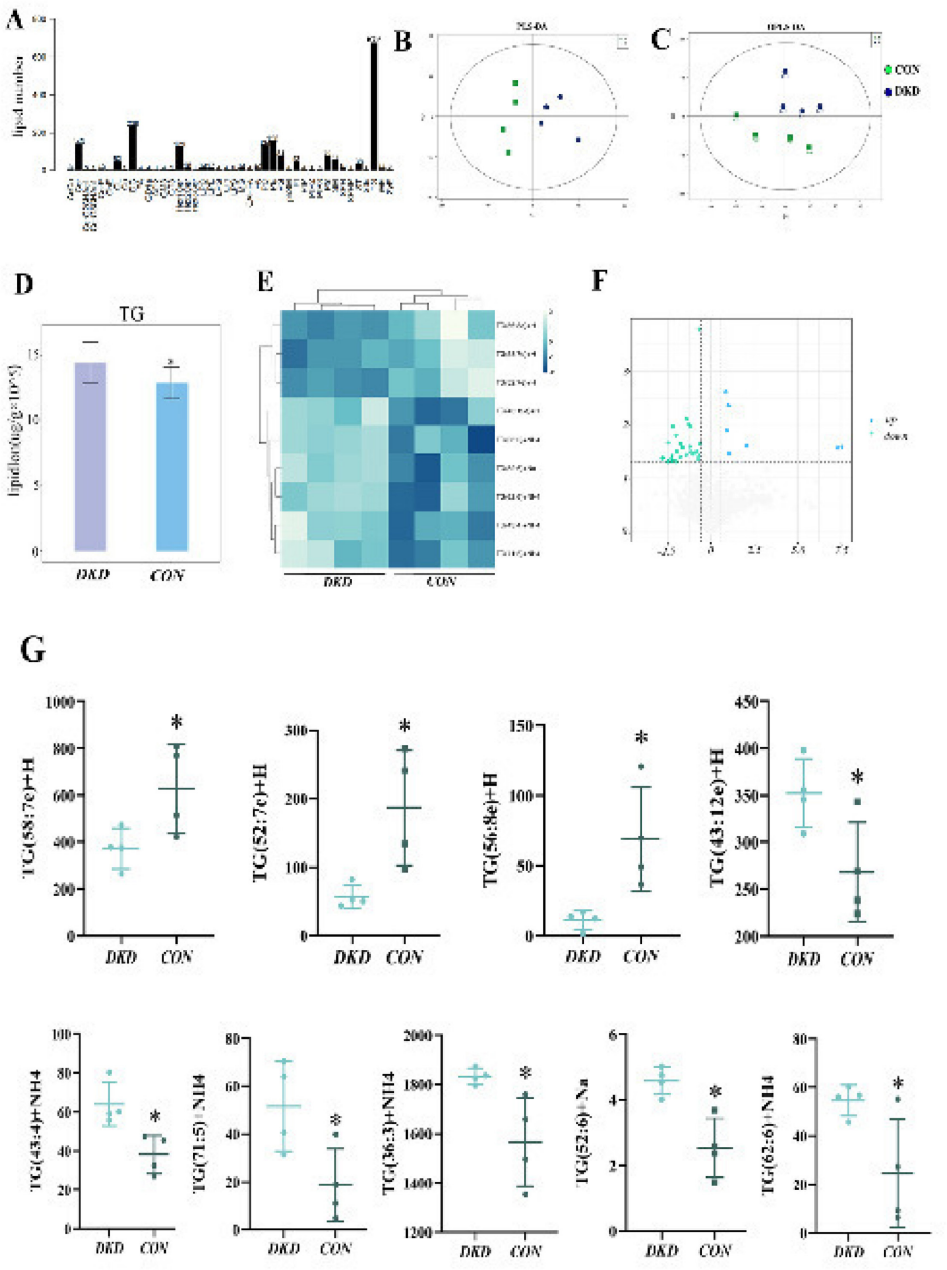
Lipidomic analysis. (A) Lipid numbers of 42 types of lipids from PRAT of two groups of mice. (B) PLS‐DA score plots of *db/db* and *db/m* mice. (C) OPLS‐DA score plots of *db/db* and *db/m* mice. (D) The TG abundance of two groups of mice. (E) Heatmap representing lipidomic features from PRAT in two groups of mice. (F) Volcano plot of the proximal and lateral regions of the PRAT in two groups of mice. (G) Individual values of the main glycerides with significant differences. *x*‐axis: DKD represents *db/db* mice, and CON represents db/m mice. The *y*‐axis represents the lipid content (μg/g). *p < 0.05.

To further understand the composition of specific lipid molecules in PRAT, we performed LC/MS‐based lipidomics analysis on two groups of mouse PRAT. We examined 8 PRAT samples, in which we identified 2022 lipid molecules among 42 kinds of lipids in the lipid spectrum. Among them, TG had the most lipid species, at 679 (Figure 3A). We used PLS‐DA (Figure 3B) and OPLS‐DA (Figure 3C) to identify the differences in metabolic characteristics between the groups in the identified data. The obvious separation between the DKD and control groups (db/m) indicated that the lipid metabolism pattern differed between the two groups. LC/MS‐based lipidomic profiling revealed TGs as the predominant lipid species in PRAT, with DKD mice exhibiting significantly higher total TG content compared to controls (Figure 3D), consistent with colorimetric assay results. The heatmap (Figure 3E) indicated that TG (43:12e)+H, TG (36:3)+NH4, TG (52:6)+Na, TG (62:6)+NH4, TG (43:4)+NH4, and TG (71:5)+NH4 were upregulated in PRAT of DKD mice, whereas TG (56:8e)+H, TG (58:7e)+H, and TG (52:7e)+H were downregulated. Figure 3G shows these specific molecules.

### 3.5 Differential Analysis of Glycerol Esters Between the Proximal and Distal Regions of PRAT in Db/Db Mice

To investigate whether there is a difference in lipid composition between the proximal and distal regions of PRAT. We analyzed the lipid spectra of the proximal (within 5 mm of the kidney) and distal regions of PRAT (beyond 5 mm from the kidney). The volcano plot revealed that 7 lipid molecules were upregulated (*p* < 0.05, FC > 1) proximal to the distal region, most of which were TGs, including TG (38:3)+NH4, TG (50:5)+NH4, TG (52:12e)+Na, TG (56:9e)+H, TG (57:6e)+H, FA (18:1)+H, and ST (m45:3)+NH4 (Figure 3F). The results revealed that the proximal region of PRAT is the main region of concentration for TGs. Among them, TG (38:3) had the most significant difference between proximal and distal PRAT in DKD mice, and it was the most abundant.

## 4 Discussion

Lipid metabolism disorders in patients with DKD exist in the early stage of renal function impairment and are characterized by increases in TG levels and lipoproteins rich in TG, including LDL, ox‐LDL, and VLDL [14]. When lipid accumulation exceeds the maximum carrying capacity of adipose tissue, lipids are deposited into nonadipose tissue, causing tissue and organ damage [15]. Renal lipid deposition is a key cause of renal lipotoxicity. Many studies have demonstrated that excessive free fatty acids (FFAs) act on podocytes, mesangial cells, and proximal tubular epithelial cells, which leads to mitochondrial dysfunction, autophagy, and endoplasmic reticulum oxidative stress, thereby activating the NF‐κB pathway and promoting glomerular fibrosis [16, 17, 18]. Moreover, ectopic deposition of lipids in the kidneys can serve as direct inflammatory mediators that activate the inflammatory response. Some inflammatory factors, such as IL‐1β and TNF‐α, stimulate macrophages in the kidney to produce reactive oxygen species (ROS). LDL oxidized by ROS, oxLDL, is engulfed by macrophages and mesangial cells, which transform into foam cells. It releases various inflammatory factors that promote extracellular matrix proliferation and lead to glomerulosclerosis. Moreover, lipid deposition further intensifies. This creates a vicious cycle. In this study, the successfully constructed DKD mouse model showed that many TGs were deposited in the kidney tissue, which effectively simulated renal lipid deposition.

Ectopic fat around the kidney can be categorized into retroperitoneal, para‐renal, peri‐renal (PRAT), and hilar fat [19]. In this study, we focused on PRAT due to its anatomical proximity to the kidney and its potential role in DKD progression.

Perirenal adipose tissue (PRAT) is enveloped by the renal fascia. When lipid deposition increases, the mechanical pressure of the renal fascia increases, which affects renal hemodynamics and decreases the renal tubular flow velocity. Continuous mechanical pressure from PRAT may activate fibrotic pathways within the kidney, leading to kidney damage and dysfunction [20]. Aldosterone is suggested to underlie the nephropathy secret in obesity not only under RASS activation but also excess caused by adipocyte‐derived aldosterone‐releasing factors [21]. Aldosterone acts via mineralocorticoid receptors (MR), leading to sodium and subsequent fluid retention by the kidney [22]. PRAT is a complex microenvironment that contains a mixture of brown and white adipocytes (BAs and WAs). White adipose tissue (WAT) can affect renal function by secreting mediators such as leptin, adiponectin, IL‐6, and TNF‐α through endocrine and paracrine activities [23].

PRF accumulation is closely related to the occurrence and development of DKD but not to non‐T2DM patients [24]. In our DKD mouse model, we cannot measure the PRF, but the weight of PRAT increased, and adipocyte expansion and TG content also significantly improved. Further detection of glomerular volume expansion, increased UACR, and increased fat deposition in the kidneys was conducted. The above results indicate a correlation between changes in PRAT and kidney injury in DKD mice, which is consistent with the findings of Lamacchia O et al. [25] Apolipoprotein C3 is present in lipoproteins rich in triglycerides and can activate NLRP3, leading to inflammation [23]. The TG increased in PRAT in our DKD mice. To investigate whether these TG activate inflammation, we detected the expression of TNF‐α in the PRAT and kidneys to indicate their inflammatory response. Our findings demonstrate that PRAT and renal TNF‐α expression were elevated in DKD mice. Beyond the inflammatory response of the kidneys, chronic inflammation of local adipose tissue is also one of the causes of DKD [26].

Research has confirmed that reducing PRAT and suppressing several proinflammatory and oxidative mediators in PRAT, including macrophage‐inflammatory‐protein‐1α (MIP‐1α), endothelin (ET‐1), 8‐isoprostane, TNF‐α, IL‐6, and IL‐1β, upregulate the heme‐oxygenase (HO) system with hemin‐normalized glycemia. This reduces renal inflammation and improves DKD in mice [27]. Our research findings provide evidence for this viewpoint. Hammoud et al. [28] reported that inflammation in PRAT induces obvious renal dysfunction before hyperglycemia and systemic inflammation in nonobese early‐stage DKD model mice. Our study also confirmed this conclusion. In PRAT, the expression of TNF‐αsignificantly differed between the two groups of mice at 12 weeks of age, and that in the DKD mice was significantly greater than that in the db/m mice. However, there was still no significant difference in kidney tissue between the two groups of mice at 20 weeks of age. These findings indicate that inflammation caused by PRAT lipid metabolism may occur before damage to the kidneys is caused by blood glucose and renal lipid deposition. These findings suggest that PRAT can be used as an early therapeutic target for DKD, delaying disease progression.

Although our study focuses on PRAT's metabolic role (producing cytokines) in DKD, other fat depots may contribute through distinct mechanisms. For instance, Hilar fat deposition and the increased retroperitoneal pressure have their largest impact where the kidney does not have a protective fibrous capsule, compressing the vascular bundle which includes the renal artery, renal vein, and lymphatic system and the nerve supply [19]. This indicates PRAT may have a similar mechanical effect on vascular/neural.

Fatty acid β‐oxidation is one of the main sources of energy for the kidney. 3‐Hydroxy‐3‐methylglutaryl‐coenzyme A synthase 2 (HMGCS2) is present in mitochondria and is the first rate‐limiting enzyme for fatty acid β‐oxidation. The increase in the level of HMGCS2 indicates that fatty acids decompose, leading to an increase in the production of ketone bodies, such as β‐hydroxybutyric (β‐OHB). HMGCS2 may play an important role in adipocyte differentiation [29, 30]. Zhao et al. have shown that HMGCS2 can promote TG synthesis and enhance energy storage in adipose tissue [31].

Although recent studies have indicated that HMGCS2 is involved in diabetic nephropathy [32], whether HMGCS2 is expressed in the PRAT and its related biological functions remain unclear. In this study, we found for the first time that HMGCS2 was expressed in PRAT and the level of HMGCS2 was significantly increased in DKD mice.

For a long time, ketone bodies have been considered a bad metabolite in diabetes because they will lead to life‐threatening diabetes ketoacidosis in patients with type 1 diabetes. However, studies have shown that upregulation of HMGCS2 expression and increased ketone body production have a protective effect on the kidneys [33, 34, 35]. The role of ketone metabolism is controversial. Our research shows that HMGCS2 gene deletion can significantly reduce fat deposition and inflammatory response in the kidneys and PRAT (especially PRAT), thereby reducing urinary protein and glomerular dilation. This finding suggests that the treatment of PRAT can focus on HMGCS2 and explore new ideas for treating DKD.

Studies have shown that not only the quantity of lipids but also the types of accumulated lipids may lead to different types of cell damage [36]. Therefore, clarifying the differences in PRAT lipids has guiding significance for subsequent research. Our nontargeted detection analysis via LC–MS revealed that TG molecules were the most abundant in PRAT and that the total TG content of PRAT in DKD mice was greater than that in control mice. The PRAT TG content was also determined via the colorimetric method, and consistent results were obtained, indicating a significant increase in TG in PRAT. The TG molecules detected in PRAT, which were mainly medium to long chains, were as follows: TG (43:12e)+H, TG (36:3)+NH4, TG (52:6)+Na, TG (62:6)+NH4, TG (43:4)+NH4, TG (71:5)+NH4. Among these molecules, TG (43:12e)+H, TG (36:3)+NH4, TG (52:6)+Na, TG (62:6)+NH4, TG (43:4)+NH4, TG (71:5)+NH4 were upregulated in DKD PRAT, while TG (56:8e)+H, TG (58:7e)+H, TG (52:7e)+H were downregulated. Our study indicates PRAT mainly consists of long‐chain triglyceride, which has the same lipid metabolism basis as dialysis patients' plasma [37].

The medial region of PRAT is located around the hilum of the kidney and is predominantly composed of brown adipocytes [38]. Many small LDs and a high density of mitochondria are stored in brown adipocytes, mainly through uncoupling protein 1 (UCP1), which results in ATP production and thermogenesis [39]. In contrast, the lateral region of PRAT is composed mainly of white adipocytes, mainly the TG [38]. We speculated that there is a difference in lipid distribution between the proximal and distal regions of PRAT. To answer this question, we conducted differential analysis on the lipid profiles of PRAT in DKD mice between the proximal (within 5 mm from the kidney) and distal (outside 5 mm from the kidney) regions. The results revealed that there were 7 upregulated TG lipid molecules in the proximal region compared with the distal region, including TG (38:3)+NH4, TG (50:5)+NH4, TG (52:12e)+Na, TG (56:9e)+H, TG (57:6e)+H, FA (18:1)+H, and ST (m45:3)+NH4. Among them, TG (38:3) was the most significantly different and abundant content between the proximal and distal ends of PPAT in DKD mice. We speculate that proximal PRAT tissue may play an important role in the occurrence and development of DKD.

## 5 Limitations

It should be noted that there are some limitations in this study. First, although we have revealed the role of HMGCS2 in regulating PRAT lipid metabolism, we cannot exclude that other regulators may also be involved in this process. Second, the molecular mechanism by which HMGCS2 regulated TG is also needed to be elucidated. In addition, the relatively small sample size of our animal experiments limits the generalizability of our findings in liposome. In future studies, we aim to expand the sample size and further investigate the underlying mechanisms to provide more robust evidence.

## 6 Conclusion

Collectively, our studies for the first time demonstrate that HMGCS2 acts as a regulator of lipid homeostasis in PRAT to promote kidney injuries and inflammation, at least in part through regulation of TG deposition.

## Author Contributions

Yuhong Huang, Miao Zeng, and Mengxue Yang conceived and directed the study. Yuhong Huang, Miao Zeng, and Lulu Jin carried out experiments. Yuhong Huang and Xiaodi Zheng analyzed and interpreted data. Yuhong Huang drafted the manuscript. Rui Zhang, Yueyue Wu, Fei Li, Bo Yang, and Jun Liu edited the manuscript. All authors approved the final version of the manuscript.

## Ethics Statement

This experimental operation was approved by the Animal Ethics Committee of the Veterinary Drug Evaluation Center of the Shanghai Institute of Veterinary Medicine, Chinese Academy of Agricultural Sciences (SV‐20230908‐07).

## Conflicts of Interest

The authors declare no conflicts of interest.

## Data Availability

The datasets generated and/or analyzed during the current study are available in the Shanghai Personal Biotechnology repository (http://www.genescloud.cn/chart/StaticVocano).
